# Arbuscular mycorrhizal fungi favor invasive *Echinops sphaerocephalus* when grown in competition with native *Inula conyzae*

**DOI:** 10.1038/s41598-020-77030-0

**Published:** 2020-11-20

**Authors:** Veronika Řezáčová, Milan Řezáč, Hana Gryndlerová, Gail W. T. Wilson, Tereza Michalová

**Affiliations:** 1 Crop Research Institute, Drnovská 507, Prague 6, Czech Republic; 2 Institute of Microbiology of the Czech Academy of Sciences, Vídeňská 1083, Prague 4, Czech Republic; 3 Department of Natural Resource Ecology and Management, Oklahoma State University, Stillwater, OK USA

**Keywords:** Ecology, Microbiology, Ecology

## Abstract

In a globalized world, plant invasions are common challenges for native ecosystems. Although a considerable number of invasive plants form arbuscular mycorrhizae, interactions between arbuscular mycorrhizal (AM) fungi and invasive and native plants are not well understood. In this study, we conducted a greenhouse experiment examining how AM fungi affect interactions of co-occurring plant species in the family Asteracea, invasive *Echinops sphaerocephalus* and native forb of central Europe *Inula conyzae*. The effects of initial soil disturbance, including the effect of intact or disturbed arbuscular mycorrhizal networks (CMNs), were examined. AM fungi supported the success of invasive *E. sphaerocephalus* in competition with native *I. conyzae*, regardless of the initial disturbance of CMNs. The presence of invasive *E. sphaerocephalus* decreased mycorrhizal colonization in *I. conyzae*, with a concomitant loss in mycorrhizal benefits. Our results confirm AM fungi represent one important mechanism of plant invasion for *E. sphaerocephalus* in semi-natural European grasslands.

## Introduction

Arbuscular mycorrhizal (AM) fungi (subphylum Glomeromycotina;^1^ are a key functional group of soil biota. AM fungi are obligate symbionts of a large majority of land plant species^2,3^, including some of the most harmful invasive species. AM fungi supply host plants with nutrients (especially phosphorus [P])^4^ and water^5^ from the soil, aid in plant pathogen protection^6,7^ and increase tolerance to drought and osmotic stresses^8–10^ in exchange for carbon [C] from the host plant^11,12^.

AM mycelium often interconnects two or more plant individuals of the same or different species, establishing arbuscular (common) mycorrhizal networks (CMNs;^13^. These CMNs play an important role in the long-distance transport of nutrients through soil ecosystems and redistributing symbiotic benefits and costs within a plant community^14^. Therefore, CMNs affect the survival, fitness, and competitiveness of their hosts, regulate plant coexistence^15–20^, and maintain plant community diversity^21^ and, therefore, ecosystem stability. Importantly, host plants have been shown to disproportionately distribute C among fungal partners according to fungal benefits (e.g., nutrient supply rates)^14,22,23^. Similarly, CMNs may distribute nutrients among plant partners according to their C supply^4,24^. The partitioning of mineral nutrients acquired via CMNs among neighboring plants and the associated C costs are likely to influence both plant competition and facilitation^19,20^.

AM fungi are, however, sensitive to perturbations that act at the ecosystem level, such as agricultural management practices, pollution (e.g., heavy metals), or plant invasion^25^. Tillage, or local distrbances, significantly impact symbiotic functioning of mycorrhiza by disrupting CMNs^26^. The subsequent reestablishment of CMNs comes at a cost for both fungi and host plants.

Plant invasions are a global phenomenon^27^ and invasive plants are a major threat to local biodiversity, community composition, and ecosystem processes worldwide^28–30^. To understand the mechanisms of invasion success of exotic plants is essential to alleviate damage caused by plant invasions. Different mechanisms of plant invasion have been postulated and most involve altered biotic interactions^31–33^, with release from natural enemies being a prominent explanation for invasive success^34^. However, as invasions are context-dependent processes, other factors such as propagule pressure, climate, time of introduction^35^, or disturbance^36^ also play a role.

The majority of studies describing underlying mechanisms for successful invasion have focused on above- rather than belowground processes, however accumulating evidence suggests soil organisms may be important regulators of plant invasions^37–40^. Although many invasive plants are mycotrophic (~ 82%^41^, and fungal associations have been shown to both facilitate and hinder invasion success^42–50^, the role of AM mycelial networks in the invasion process has not been determined. Further, information on the role of mycorrhizae on invasive plant success is available for only a small number of plant species at this time.

Two main hypotheses have been proposed to explain the role of mycorrhizal fungi in plant invasions, both of which are based on the invasive plants interacting differently with AM fungi relative to native plants: (i) the ‘*degraded mutualism hypothesis*’^51^ and (ii) the ‘*enhanced mutualist hypothesis*’^52^. The degraded mutualism hypothesis indicates invasive plants either do not form AM (e.g., Brassicaceae or Proteaceae), or are poorly colonized with low dependency on AM fungi in its new range, thereby suppressing AM fungal abundance. By doing so, invasive plants strongly affect mycorrhizal symbiosis of native mycorrhizal plants, often reducing native plant competitiveness^46,53^. (ii) The ‘*enhanced mutualist hypothesis*’^52^ indicates invasive plants receive greater benefit from the symbiosis than native plants, altering native AM fungal communities and increasing invasive species competitiveness^50,52,54,55^. Therefore, invasive plants in their new range may parasitize local CMNs, deriving disproportionally large benefits compared to their symbiotic costs at the expense of competing native plants. CMNs have been shown to preferentially transfer mineral nutrients (^15^N and P) to an invasive plant, with less transferred to the native species^56^. CMNs mediation of invasive and native plants may be crucial to the understanding of invasion success or naturalization of an invasive plant and concomitant ‘spread*’*: these aspects of invasion have not currently been widely studied.

**Figure 1:**
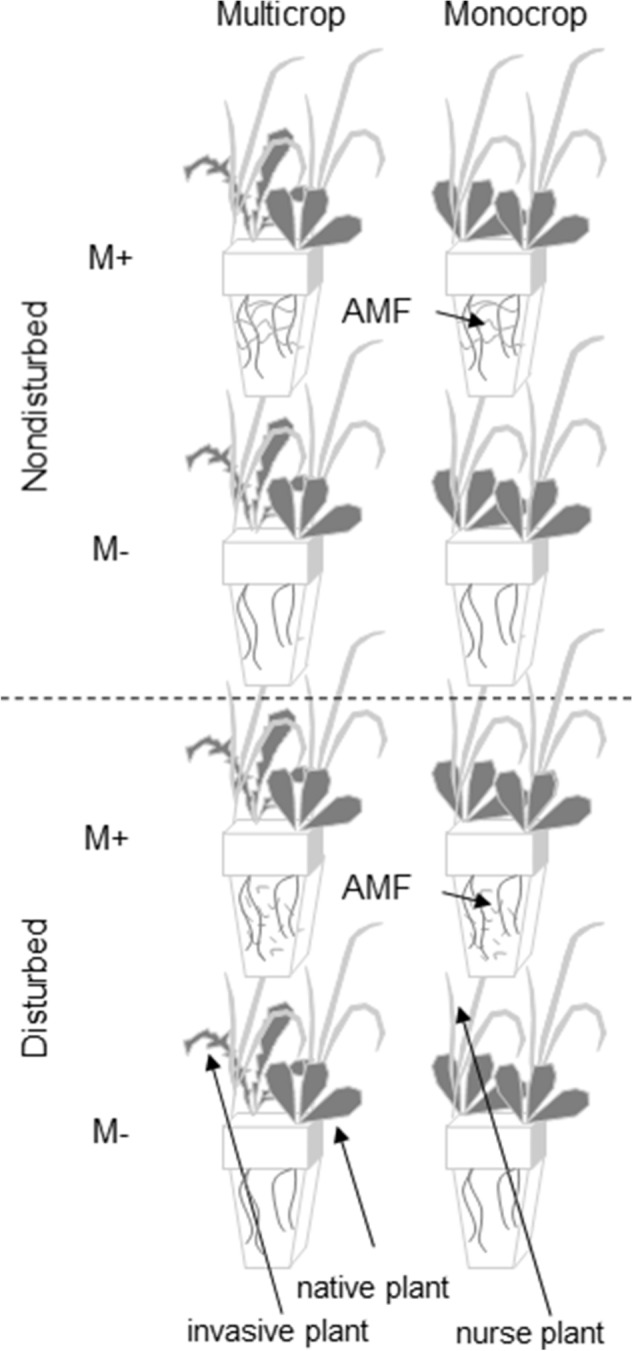
Experimental design. This design was used for both harvests. Both mycorrhizal (M+) and nonmycorrhizal (M−) pots were pre-planted with a nurse plant *Festuca pratensis*. Soil was disturbed or left intact before target plants were planted, resulting in disturbed or non-disturbed arbuscular mycorrhizal (AM) networks in M+ treatment. Each figured pot contained 5 replicates.

Because it has been shown that the majority of invasive plants are mycotrophic^41,57,58^ and able to establish mycorrhizal associations in the secondary range^49^, our current study will focus on the ‘*enhanced mutualism hypothesis’*. We selected mycorrhizal plant species *Echinops sphaerocephalus* commonly invasive to central Europe and conducted a coexistence (intercropping) experiment to determine if feedbacks between AM fungi, invasive plant species, and native plant species (*Inula conyzae*) play a role in successful invasions by the non-native (Fig. 1). We hypothesized that (i) presence of AM fungi enhances success of a mycotrophic invader in competition with a domestic plant—AM fungi preferentially support plant growth and nutrition of the invasive plant, with a concomitant reduction in native plant growth and nutrition, (ii) competitive advantage of the mycotrophic invader provided by AM fungi is in initial growth phases more pronounced in non-disturbed than in disturbed environments, where (compared to non-disturbed environment) it increases with time.

## Results

### AM fungal development

**Figure 2:**
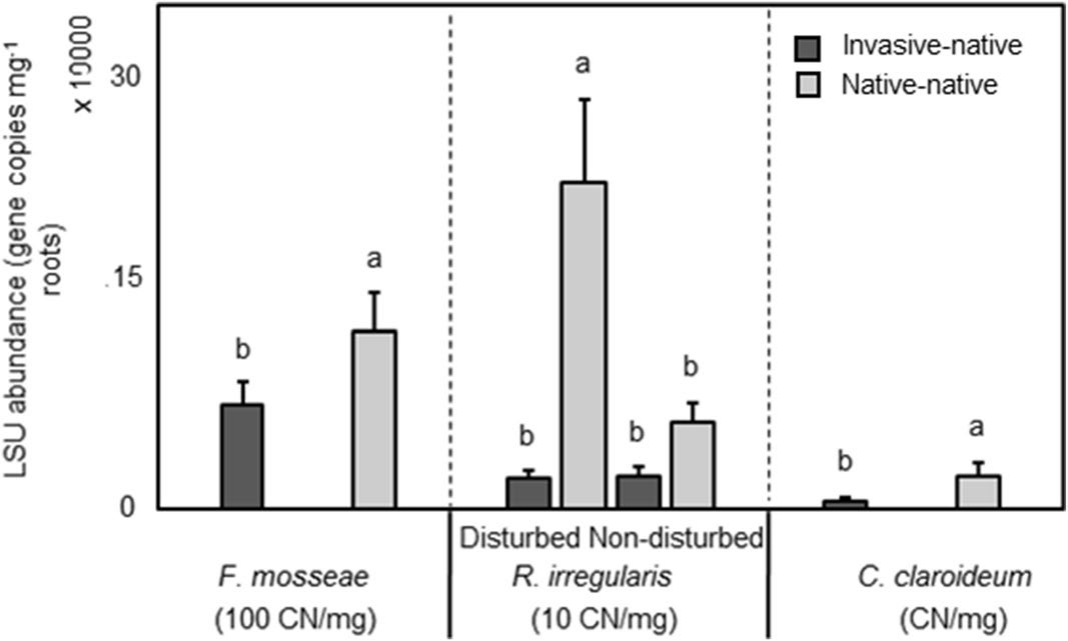
Abundance of the different arbuscular mycorrhizal fungal taxa in the roots of the native plant species *Inula conyzae* as affected by community assembly i.e., plant combination (invasive-native: invasive and native plant growing in competition; native-native: only native plants of the same species growing together) or community assembly and soil disturbance (disturbed: substrate in the pot disturbed before target plants inserted; non-disturbed: target plants planted into non-disturbed substrate, resulting in non-disturbed CMNs of M+ pots) after the second harvest. Bars represent means accompanied by standard errors (n = 20 or n = 10, respectively). Different letters above individual bars indicate significant differences between means at *P* < 0.05.

While AM fungal structures were present in roots of all M+ plants (Supplementary Table S6), microscopic observation confirmed that no root samples of M− plants contained AM fungal structures. Following the second harvest, the abundances of *F. mosseae*, *C. claroideum*, and *R. irregularis* in the roots of *I. conyzae* were significantly (*P* = 0.033, 0.011, 0.005 × 10^−2^, respectively) lower when grown with the invasive (multicrops) (7,164,400 ± 1,610,100 CN mg^−1^, 5420 ± 2869 CN mg^−1^, 196,989 ± 45,104 CN mg^−1^, respectively), compared to monocrops (12,312,900 ± 2,696,900 CN mg^−1^, 23,999 ± 9348 CN mg^−1^, 1,142,609 ± 340,826 CN mg^−1^, respectively; Fig. 2. However, in the case of *R. irregularis,* this difference was significant only following disturbance Fig. 2, Supplementary Table S3).

Disturbance consistently increased the relative abundance of *R. irregularis* in *I. conyzae* roots (from 302,508 ± 95,731 CN mg^−1^ to 1,375,367 ± 435,243 CN mg^−1^; *P* = 0.004), however, the increase was significant only in monocrop pots Fig. 2, Supplementary Table S3).

The abundances of individual AM fungal taxa were not significantly different in roots of invasive *E. sphaerocephalus* or its paired *I. conyzae* (Supplementary Fig. S1). Disturbance did not significantly affect the abundances of any AM fungal taxa (Supplementary Fig. S1). The abundances of AM taxa were generally decreased (3,209,666 ± 479,223 CN mg^−1^ and 201 ± 196 CN mg^−1^ for *F. mosseae* and *C. claroideum*, respectively) at the second harvest, compared to the first (8,713,356 ± 1,055,110 CN mg^−1^ and 15,447 ± 4092 CN mg^−1^ for *F. mosseae* and *C. claroideum*, respectively; Supplementary Fig. S1), with the abundance of *R. irregularis* in roots of *I. conyzae* as the only exception (Supplementary Fig. S1).

### Competition for resources between the invasive and paired native plant

There were no significant differences between biomass of nurse plants in monocrop (native-native) or paired (native-invasive) treatments. Nurse plants did not re-grow following harvest.

**Figure 3:**
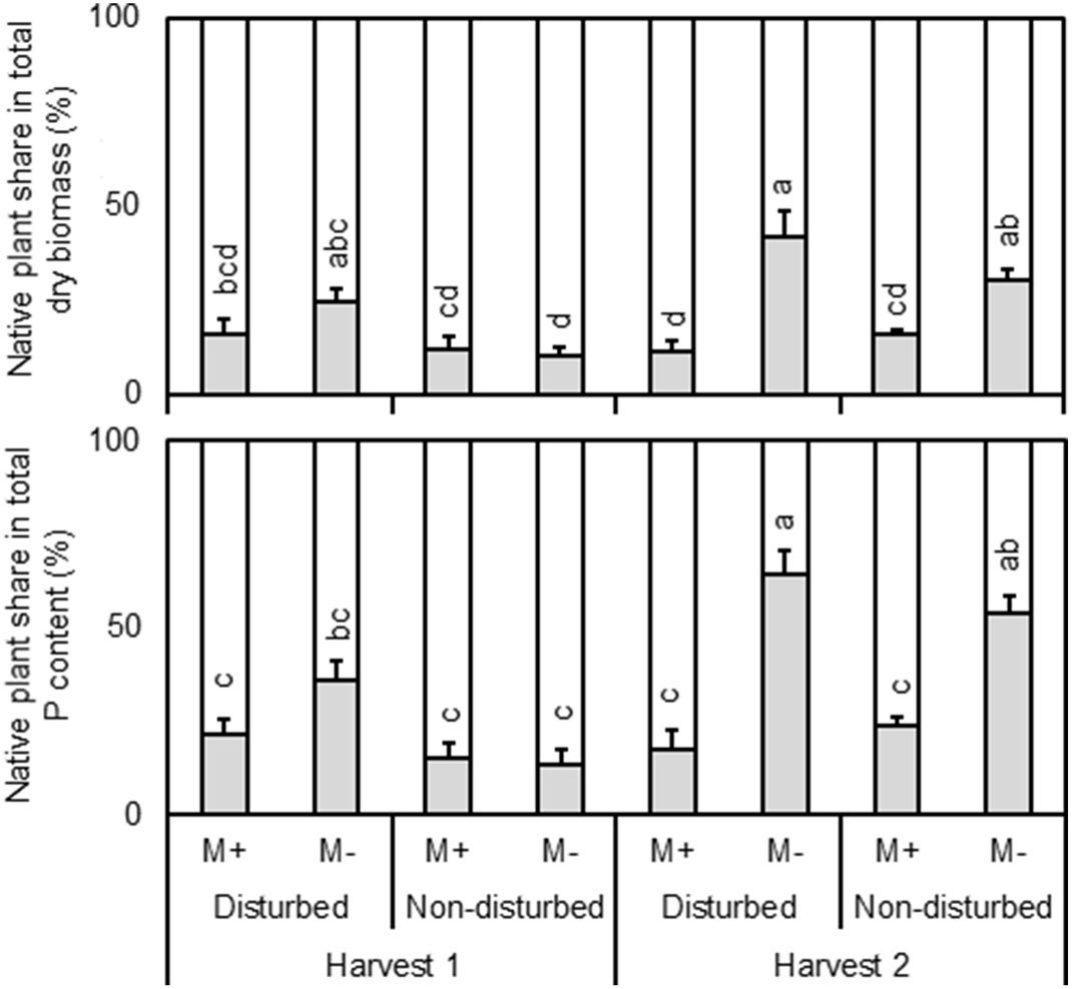
Fraction of shoot dry biomass and fraction of shoot P content of native *Inula conyzae* (gray box), growing in pairs with invasive *Echinops sphaerocephalus* (white box) detected in the plant biomass per cultivation pot (i.e., the share of resources diverted to the native plant on a whole cultivation pot basis, with the remaining part of the particular resource being assignable to the invasive plant) as affected by mycorrhizal inoculation (M+: mycorrhizal inoculum added; M−: nonmycorrhizal control), initial soil disturbance (disturbed: substrate in the pot disturbed before target plants inserted; non-disturbed: target plants planted into non-disturbed substrate, resulting in non-disturbed CMNs of M+ pots), and harvest (harvest 1: first harvest; harvest 2: second harvest). Bars represent means accompanied by standard errors (n = 5). Different letters above individual bars indicate significant differences between means at *P* < 0.05.

To assess competition between the two coexisting plant species, we calculated the fraction of total plant biomass and P content of each native plant (i.e., the relative biomass, or ‘share’ of resources, diverted to the native plant on a whole pot basis, with the remaining portion of the particular resource assumed to belong to the invasive plant). Based on this calculation, both relative biomass production and P content of native *I. conyzae* consistently decreased (from 26 and 42% to 14% and 20%, respectively) when inoculated with mycorrhizal fungi (*P* = 0.004 and *P* = 0.0003, respectively; Fig. 3, Supplementary Table S4). However, there were significant interactions between mycorrhizal inoculation and harvest Fig. 3, Supplementary Table S4). These interactions reflected the absence of significance between M− and M+ plants at the first harvest, compared to significantly (*P* = 0.004 and 0.0003, respectively) greater values for M− plants of the second harvest Fig. 3. Relative biomass production and P content of nonmycorrhizal (M−) *I. conyzae* consistently increased following disturbance (from 20 ± 4% and 34 ± 7% to 33 ± 5% and 50 ± 6%, *P* = 0.02 and 0.01, respectively, Fig. 3, Supplementary Table S4).

### Changes in plant biomass and mineral nutrition of native I. conyzae associated with competition

**Figure 4:**
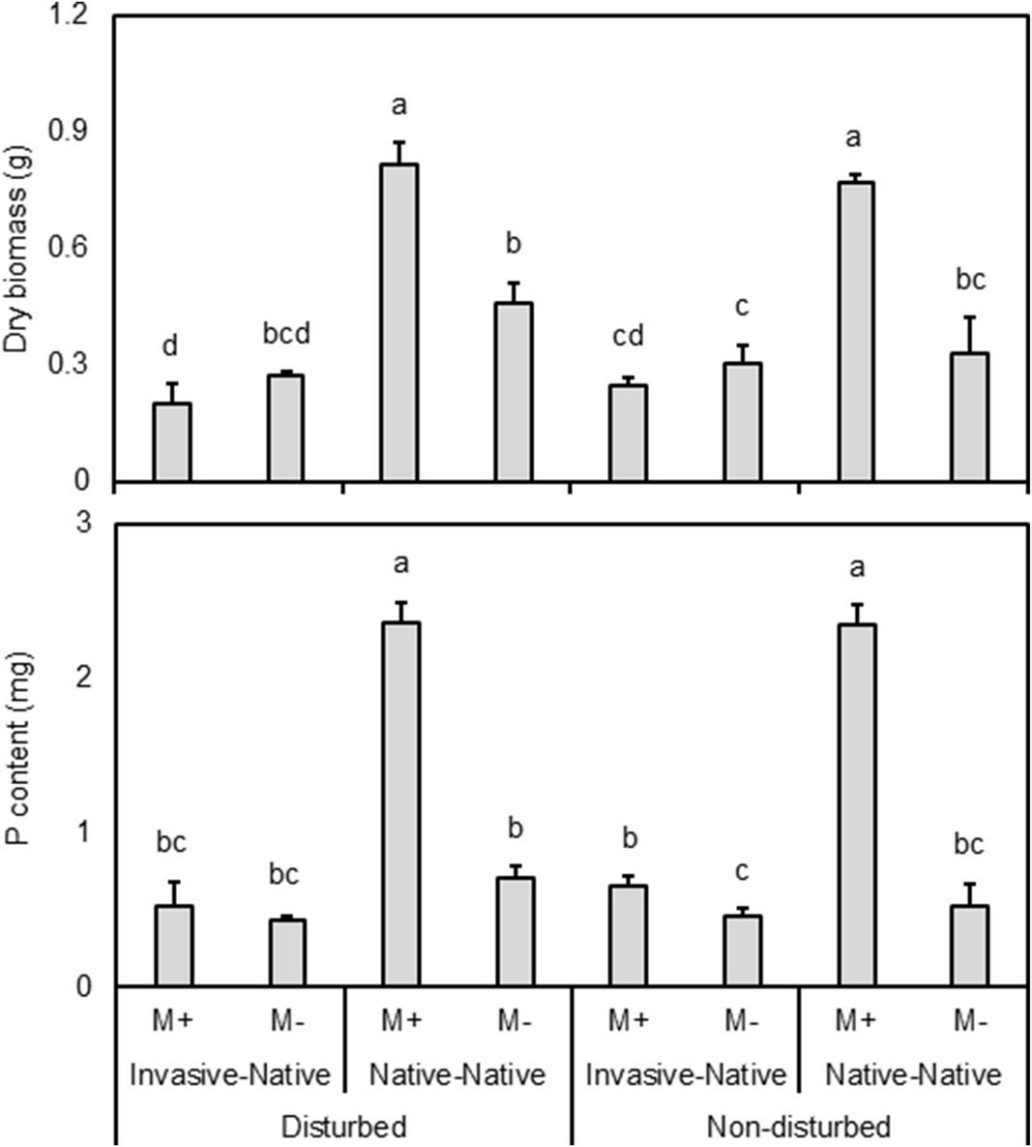
Shoot dry biomass and shoot P content of the native plant species *Inula conyzae* following the second harvest, as affected by mycorrhizal inoculation (M+: mycorrhizal inoculum added; M−: nonmycorrhizal control), community assembly i.e., plant combination (invasive-native: invasive and native plant growing in competition; native-native: only native plants of the same species growing together) and initial soil disturbance (disturbed: substrate in the pot disturbed before target plants inserted; non-disturbed: target plants planted into non-disturbed substrate, resulting in non-disturbed CMNs of M+ pots) after the second harvest. Bars represent means accompanied by standard errors (n = 5). Different letters above individual bars within vertical dashed lines indicate significant differences between means at *P* < 0.05.

Following the second harvest, plant biomass of native plant *I. conyzae* was consistently greater (compare 0.86 ± 0.06 g and 0.45 ± 0.09 g) following mycorrhizal inoculation when grown without the invasive *E. sphaerocephalus*, however mycorrhizal inoculation generally had no effect on *I. conyzae* biomass when grown with the invasive plant Fig. 4, Supplementary Table S5). As a result, there were significant interactions between mycorrhizal inoculation and community assembly (*P* = 0.001 × 10^−1^).

Community assembly generally decreased plant biomass of *I. conyzae* (from 0.66 ± 0.07 g to 0.25 ± 0.02 g; *P* = 0.007 × 10^−4^). However, this decrease was significant only in M+ plants Fig. 4. P content of native *I. conyzae* was also consistently (*P* = 0.001 × 10^−3^) greater in mycorrhizal plants (1.6 ± 0.2 mg), compared to those without mycorrhizal inoculation (0.6 ± 0.1 mg), except in the multicrop following disturbance, where the effect of mycorrhizal inoculation was not observed Fig. 4. P content of native *I. conyzae* was consistently (*P* = 0.003 × 10^−4^) lower (0.5 ± 0.1 mg) when grown with the invasive, compared to monocrop pots (1.7 ± 0.3 mg), however, the decrease was significant only in M+ pots Fig. 4.

The results of the first and second harvest were similar for both plant biomass and P content, however significant trends only emerged in the second harvest. Therefore, results of the first harvest are reported in the Supplementary Fig. S2.

## Discussion

In agreement with our hypothesis which predicted AM fungi help facilitate the success of a mycotrophic invasive plant in competition with a domestic plant, our study shows that there were negative effects of AM fungi on proportional total biomass and P content of native *I. conyzae*. Our results indicate that the presence of AM fungi enhanced the competitive ability of invasive *E. sphaerocephalus* against native *I. conyzae*. This is in agreement with Callaway et al.^59^, and Workman and Cruzan^60^ who reported positive effects of AM fungi on biomass production of invasive *Centaurea melitensis* and *Brachypodium sylvaticum*, respectively, growing in competition with native plants.

Contrary to our second hypothesis, the difference between the M+ and M− treatments was more pronounced in plants grown in experimentally disturbed soil. In fact, the positive effect of AM fungi abolished negative effects of disturbance on the invasive *E. sphaerocephalus*. This hypothesis was based on our assumption that competitive advantages of invasive plant would originate from linking with existing CMNs and disproportionally profit, at the expense of competing native plants, with the strongest advantage observed in invasive plants grown in pots with non-disturbed CMNs. However, this was not supported by our data, as the invader was competitively more proficient with mycorrhizal inoculation in both initially disturbed and non-disturbed treatments. Although we cannot confirm or reject existence of CMNs directly, molecular analyses indicated that both the invasive and native plants generally shared the same AM fungal taxa, with *F. mosseae* being dominant, regardless of native or invasive plant species. The absence of effects of disturbance on abundance of AM fungi supports a rapid recovery of CMNs following disturbance. Therefore, the invasive plant with intact mycelium was not at an advantage over invasive plants with initially disturbed mycelium.

The disproportionate distribution of mycorrhizal benefits by CMNs between these invasive and native plants may have played a role in the competitive success of invasive *E. sphaerocephalus* grown with domestic *I. conyzae*. However, the competitive advantage of *E. sphaerocephalus* over *I. conyzae* is more likely due to lower abundances of AM fungal taxa in roots of *I. conyzae*, when grown in the presence of invader (*E. sphaerocephalus*), compared to growth without the invasive. Our results are similar to Zhang et al.^61^, where invasive *Solidago canadensis* inhibited AM fungal root colonization of native species. Callaway et al.^62^ found the invasive plant *Alliaria petiolata* suppressed native AM fungi, resulting in an indirect inhibition of native mycorrhizal plants.

The mechanisms of mutualist degradation may be mediated by allelochemical production by invasive plants. Allelopathy likely played a role in the invasion success of non-native *Echinops echinatus* when grown with native *Argemone mexicana*^63^. While beyond the scope of our current study, it is possible that allelopathic biochemicals produced by invasive *E. sphaerocephalus* decreased mycorrhizal colonization in native *I. conyzae*, thereby reducing mycorrhizal benefits. However, AM fungi continued to be beneficial to *E. sphaerocephalus*. Therefore, it is likely that the decrease in abundance of AM fungi in the roots of domestic *I. conyzae* when grown with the invasive plant is a reflection of root turnover, as colonized roots died and were replaced by new root growth not able to form associations with the fungal symbiosis. This was also reflected by reduced biomass production of the native plant when grown with the invasive plant.

## Conclusions

In our current study, we focused on competition of important invasive species from the family Asteraceae in central Europe with a co-occurring native forb, both are abundant in invaded semi-natural plant communities. Among the economically and ecologically important mycotrophic invasive plants in Central Europe, the Asteraceae family is the most abundant. Additionally, we selected species from one family (Asteraceae), as domestic plants of the same family typically bring the least bias.

The effects of AM fungi on invasive plant growth and P status recorded in our study indicate that AM fungi can play an important role in invasive plant competitive success. However, decreases in abundance of AM fungi followed by decreases in mycorrhizal benefits in *I. conyzae* growing in competition with invasive *E. sphaerocephalus* do not support the ‘*enhanced mutualist hypothesis*’ but instead point to the ‘degraded mutualism hypothesis’.

Ecosystems containing nonnative invasive plant species are common, but mechanisms promoting their co-occurrence are not well understood. It may become increasingly important to study the widespread effects of AM fungi on nonnative plant invasibility and establishment as these fungi affect plant species coexistence and community composition^64–66^. Understanding the mechanisms leading to successful invasion may be especially important in light of global alterations such as increases in invasive plant species, but also climate change, alterations in nutrient availability, and land use changes. This is the first study to directly assess the role of AM fungi in the competition of *E. sphaerocephalus* with a native plant. Our experiment was limited to pair-wise interactions among plant species, and this is a critical first step in resolving complex interactions that occur among native and nonnative plant species in a community. The next step will include assessments of additional invasive-native plant pairs to allow generalization of the results to a broader range of plant taxa, with an ultimate goal of assessing AM fungi in field studies.

## Materials and methods

### Experimental design

The experiment was a fully factorial design with four factors: (1) mycorrhizal inoculation (inoculated with mycorrhiza, M+; or not, M−), (2) community assembly (monocrop = native plant paired with native plant; multicrop = native plant paired with invasive plant), (3) soil disturbance (no disturbance; initially mechanically disturbed), and (4) harvest (harvest 1 or harvest 2; Fig. 1. There were five replicate pots established per treatment combination, for a total of 80 pots that were completely randomized.

### Cultivation pots and substrate

Plants were grown in 2-L pots (11 × 11 × 20 cm, w × d × h) lined with a plastic mesh (1.2 mm opening) at the bottom, sterilized with 96% ethanol and filled with a potting substrate. The substrate consisted of thoroughly mixed (volume-based) 10% γ-irradiated (> 25 kGy) field soil from Litoměřice, Czechia (N50°31′54.53″ E14°06′7.10″), 45% autoclaved zeolite MPZ 1–25 from Zeopol (www.zeolity.cz, grain size 1–2.5 mm), and 45% autoclaved quartz sand (grain size < 3 mm). For physicochemical properties of the substrate see^67^ or Supplementary Table S1.

### Mycorrhizal inoculation

Half of the pots (M+) were supplemented with 36 g of mycorrhizal inoculum. The inoculum consisted of potting substrate containing root fragments of leek (*Allium porrum* L.), which had been used as a host plant in previous pot cultures of *Rhizophagus irregularis* (N.C. Schenck & G.S. Sm.) C. Walker & Schuessler (2010) BEG 158, *Claroideoglomus claroideum* (N. C. Schenck & G. S. Sm.) C. Walker & Schuessler (2010) BEG 155, and *Funneliformis mosseae* (T.H. Nicolson & Gerd.) C. Walker & Schuessler (2010) BEG 161. BEG is an abbreviation for the International Bank for the Glomeromycota (www.i-beg.eu). The three monospecific inocula were mixed in the volume ratio 1:1:1 (*v*:*v*:*v*). The other half of the pots (M−) received 36 g nonmycorrhizal (mock) inoculum. The mock inoculum consisted of potting substrate containing root fragments of leek from a previous pot culture grown under the same conditions and for the same period of time as the M+ pot cultures (above) but without AM fungi. The inocula were added 4–5 cm beneath the surface of the potting substrate.

### Plants

One pair of invasive-native mycorrhizal herbaceous plant species (family Asteraceae) was used in this study. We selected *Echinops sphaerocephalus* L., a common invader of semi-natural plant communities in central Europe (Czechia), and *Inula conyzae*, an indigenous herb which occurs abundantly in invaded plant communities. Prior to planting native and invasive species, both M+ and M− treatments were preplanted with *Festuca pratensis* Huds. *F. pratensis* served as a nurse plant to establish CMNs in M+ pots that were not associated with either the invasive or native plant species. All seeds were field collected. To account for possible genotypic differences among individuals, we selected seeds from at least 10 individuals per plant species of few plant populations, mixed the seeds and randomly distributed among different treatments. Seedlings were pre-germinated for two weeks in Petri dishes on wet filter paper at room temperature. Pots were directly sown by seeds of *F. pratensis* in the middle of January. After 48 days, half of the pots were once mechanically disturbed by inserting a long metallic spatula to its full depth into the central part of the pot (from one empty corner to the other avoiding nurse plants). Microscopic assessment of soil samples (following^68^ of 6M+ and 2M− pots 48 days post disturbance conferred hyphal length density in the potting substrate comparable to similar experiment^69^. Target seedlings were transplanted into pots (two individuals per pot—either two *I. conyzae*, or one *I. conyzae* paired with one *E. sphaerocephalus*) immediately after soil disturbance. Target plants (*E. sphaerocephalus* and *I. conyzae*) were planted into the two empty corners of each pot (i.e. corners not occupied by *F. pratensis*) Fig. 1. Target plants in M+ treatments were planted into non-disturbed soil with intact CMNs, or into initially disturbed soil, where CMNs needed to reestablish from spores and hyphal fragments. Thirty-six days following the initial disturbance and planting of target plants, shoots of all nurse plants were cut under the hypocotyl–root interface and dried for 3 days at 65 °C and weighted.

### Growth conditions

Target plants were grown spring (March–May) 2018 in a glasshouse at the Institute of Microbiology, Prague, with average day and night temperatures 24 °C and 20 °C, respectively. The day length was extended to 12 h with supplemental lighting (metal halide lamps, 250 W each) providing a minimum photosynthesis flux density of 200 μmol m^−2^ s^−1^. Plants were watered daily. From the fourth week after planting *F. pratensis*, each pot received weekly 65 ml of Long Ashton mineral nutrient solution^70^ with the P concentration reduced to 20% of the original recipe^67,71^.

### Plant harvest

The experiment consisted of two harvest times (64 and 91 days after target plants were planted) to assess the effects of disturbance across time. The shoots of all target plants were cut at the hypocotyl–root interface and subsequently dried for 3 days at 65 °C to determine shoot dry weight (hereafter called plant biomass). The roots were washed from the substrate under cold tap water, weighed, and cut into 1.5 cm fragments. The roots were then divided into three sections. One section was immersed in 50% ethanol to determine AM fungal colonization. The second was kept in the freezer at − 20 °C for molecular analyses. The last section was weighed, dried, and weighed again; fresh to dry weight ratio was determined and total root dry weight was calculated for each root system.

### Analyses and calculations

To evaluate mycorrhizal benefits provided by AM fungi to the plant, shoot dry biomass and P content were assessed. To determine P mass fraction in aboveground plant tissues, 100 mg of a milled sample of each shoot was incinerated in a muffle furnace at 550 °C for 12 h, the ashes were dissolved in 1 ml of concentrated (69%, w:v) HNO_3_ and briefly boiled (250 °C) on a hot plate. The extracts were then transferred into volumetric flasks (50 ml) through ashless filter paper (Whatman 41, P-lab, Prague, Czechia) and ultrapure water added for a final volume of 50 ml. Orthophosphate concentration in the extracts was measured using the malachite green method^72^. P content per shoot (hereafter referred as plant P content) was calculated from the measured nutrient mass fraction in shoots using the dry biomass of the shoots.

The absence of AM fungal structures in all M− roots was checked microscopically (one composite sample per M− pot) using the magnified intersection method by McGonigle et al.^73^ after staining the roots with trypan blue^74^, [with minor modifications]. Roots from each collected sample were cut into 2-cm-long segments and placed in processing cassettes (customized scintillation vials). Root pieces were cleared in 10% KOH at 80 °C for 30 min in a water bath. Cleared pieces of roots were rinsed with tap water to remove KOH, and roots were immersed in 1% HCl at room temperature for 30 min followed by heating at 80 °C for 15 min. Roots were rinsed with tap water and stained with 0.05% trypan blue by incubation at 30 °C for 30 min. Root fragments were then transferred to vials containing lactoglycerol to allow excess stain to leach from roots. Stained root samples were stored in lactoglycerol solution for at least 48 h before being mounted on microscopic slides. One hundred root intersections were scored per sample.

To assess AM fungal abundance in roots of M+ plants, DNA was extracted from frozen roots (70–80 g per sample) by the glass milk method with the CTAB extraction buffer as described in Gryndler et al.^75^ with minor modifications (the samples were frozen in the CTAB buffer before homogenization, a wash buffer was applied twice, the samples were incubate 5 min in 65 °C during elution). An internal standard was added to the samples before DNA extraction to correct subsequent real-time PCR (qPCR) analyses for DNA loses upon extraction and for PCR inhibition^76^. To this end, 2 × 10^10^ copies of the linearized plasmid carrying fragment of cassava mosaic virus DNA (GenBank accession number AJ427910) were used, and recovery after the extraction was quantified by qPCR. The DNA recovery rates of the internal standard for each individual DNA sample (15.2 ± 0.9 in this study) were used to correct the qPCR results obtained with the AM fungal taxa‐specific markers, as described in von Felten et al.^77^. The qPCR was further used to assess abundance of the three AM fungal taxa in roots using taxon-specific primers and hydrolysis (TaqMan) probes targeting the nuclear large ribosomal subunit (nLSU) gene (^76^, Supplementary Table S2), HOT FIREPol Probe qPCR Mix Plus(ROX) chemistry (Solis BioDyne, Tartu, Estonia) and StepOnePlus qPCR Cycler (Applied Biosystems, now Thermo Fisher Scientific, Waltham, Massachusetts). The DNA extracts were diluted 10 × before the qPCR. The reaction conditions and calculations followed Janoušková et al.^78^. Only M+ samples were analyzed, two DNA extracts from M− pots were used as a negative control.

### Statistical analyses

Analyses of variance (ANOVA) with *P* < 0.05 as the significance cutoff level were calculated in the R 3.6.3 statistical environment (R Core Team, 2013, https://www.R-project.org/) after checking for data conformity with ANOVA assumptions (i.e., normality and homogeneity of variances). Plant biomass and plant P content were log-transformed before the analyses. Three-way ANOVAs with factors mycorrhizal inoculation, community assembly and soil disturbance were performed on biomass and plant P content of the native plant species either growing in monocrops or paired with the respective invasive plants. The average biomass and P content of monocrops per cultivation pot were used when assessing the effect of plant invasion on native plants biomass. Three-way ANOVAs with factors mycorrhizal inoculation, soil disturbance and plant harvest were performed on native plant share (i.e., fraction of the total assignable to the native plant contribution) in the per-pot summed values of total plant biomass and plant P content. Three-way ANOVAs with factors community assembly, soil disturbance and harvest were performed on AM fungal taxon abundances (measured by qPCR) of the native plant species. Three-way ANOVAs with factors plant species, soil disturbance, and harvest were performed on AM fungal taxon abundances of the native plant species and paired invasive plant. When appropriate, post-hoc comparisons were carried out using Tukey HSD tests. Mean values and standard errors per treatment combination are presented.

## Supplementary information

Supplementary Tables.
