# Supplementary material for: Arbuscular mycorrhizal fungi favor invasive Echinops sphaerocephalus when grown in competition with native Inula conyzae
Source: Sci Rep. 2020 Nov 20;10:20287. doi: 10.1038/s41598-020-77030-0 (PMC7679399; doi:10.1038/s41598-020-77030-0)
Supplement: Supplementary file 1 — Supplementary Tables. [file 41598_2020_77030_MOESM1_ESM.pdf]

Supplementary Material

Arbuscular mycorrhizal fungi favor invasive *Echinops sphaerocephalus* when grown in competition with native *Inula conyzae*

Veronika Řezáčová[1,2,*], Milan Řezáč[1,2], Hana Gryndlerová[1,2], Gail W. T. Wilson[3], and Tereza Michalová[2]

[1] Crop Research Institute, Drnovská 507, Prague 6, Czech Republic

[2] Institute of Microbiology of the Czech Academy of Sciences, Vídeňská 1083, Prague 4, Czech Republic

[3]Department of Natural Resource Ecology and Management, Oklahoma State University, Stillwater, OK, USA

*Author for correspondence: Veronika Řezáčová; Tel: +420 241 062 382; Email: rezacova@vurv.cz; ORCID: 0000-0002-1749-0355

TABLE S1 Physicochemical properties of potting substrate mixed from previously sterilized components (soil : zeolite : sand, 10 : 45 : 45, v: v: v). pH (pH_{water}) was measured in aqueous soil suspension (1 : 2.5, w : v) after shaking for 1 h. Total phosphorus (P) was assessed colorimetrically following incineration of the substrate at 550°C and extraction with hot 14 M HNO_3. Water-extractable P was measured colorimetrically in aqueous soil extract (1 : 10, w : v) after shaking the suspension for 20 h and filtration through 0.2 µm membrane filter. Total nitrogen (N) and organic carbon (C) as well as isotopic composition of the C were assessed using a Flash EA 2000 elemental analyzer coupled with a Delta V Advantage isotope ratio mass spectrometer (Thermo Fisher Scientific, Waltham, MA, USA). Isotopic composition of the C is given with respect to the international Vienna Pee Dee Belemnite (VPDB) standard.

	mean value of 3 analytical reps
pH_{water}	8.90
Total P (mg kg^{-1})	46.5
Water-extractable P (mg kg^{-1})	2.95
Total N (%)	0.01
Total organic C (%)	0.22
$\delta^{13}C$ (vs. VPDB standard, in ‰)	−18.11

TABLE S2 Sequences of primers and hydrolysis probes (sequences (5′→3′)) used for the quantitative real-time PCR quantification of large ribosomal subunit gene copies of the different arbuscular mycorrhizal AM fungal taxa as described in [1].

	R. irregularis	*C. claroideum*	*F. mosseae*
Forward primer	TTCGGGTAATCAGCCTTTCG	GCGAGTGAAGAGGGAAGAG	GGAAACGATTGAAGTCAGTCATACCAA
Reverse primer	TCAGAGATCAGACAGGTAGCC	TTGAAAGCGTATCGTAGATGAAC	CGAAAAAGTACACCAAGAGATCCCAAT
Hydrolysis probe	TTAACCAACCACACGGGCAAGTACA	AACAGGACATCATAGAGGGTGACAATCCC	AGAGTTTCAAAGCCTTCGGATTCGC

TABLE S3 Significances of the effects of inoculation, disturbance, harvest and their interactions as revealed by three-way ANOVA (n = 5) on fraction of shoot dry biomass and fraction of shoot P content of native *Inula conyzae*, growing in pairs with invasive *Echinops sphaerocephalus* detected in the plant biomass per cultivation pot (i.e., the share of resources diverted to the native plant on a whole cultivation pot basis, with the remaining part of the particular resource being assignable to the invasive plant). Asterisks denote the levels of statistical significance as follows: ***, $P < 0.001$; **, $0.001 \leq P < 0.01$; *, $0.01 \leq P < 0.05$; ns, $P \geq 0.05$.

	Dry biomass	P content
Inoculation	***	***
Disturbance	*	*
Harvest	**	***
Inoculation x disturbance	*	*
Disturbance x harvest	ns	ns
Inoculation x harvest	**	***

TABLE S4 Significances of the effects of inoculation, community assembly, disturbance and their interactions as revealed by three-way ANOVA (n = 5) on shoot dry biomass and shoot P content of the native plant species *Inula conyzae* following the second harvest. Asterisks denote the levels of statistical significance as follows: ***, $P < 0.001$; ns, $P \geq 0.05$.

	Dry biomass	P content
Inoculation	ns	***
Community assembly	***	***
Disturbance	ns	ns
Inoculation x community assembly	***	***
Community assembly x disturbance	ns	ns
Inoculation x disturbance	ns	ns

TABLE S5 Significances of the effects of community assembly, disturbance and their interactions as revealed by two-way ANOVA (n = 5) on abundance of the three AM fungal taxa in the roots of experimental plants following the second harvest and measured using quantitative real-time PCR with taxon-specific molecular markers. Asterisks denote the levels of statistical significance as follows: ***, $P < 0.001$; **, $0.001 \leq P < 0.01$; *, $0.01 \leq P < 0.05$; ns, $P \geq 0.05$.

	Funneliformis mosseae	*Claroideoglomus claroideum*	*Rhizophagus irregularis*
Community assembly	*	*	***
Disturbance	ns	ns	**
Community assembly x disturbance	ns	ns	**

Fig. S1 Abundance of the different arbuscular mycorrhizal fungal taxa in the roots samples of the native plant species *Inula conyzae* and invasive plant *Echinops sphaerocephalus* as affected by plant species (different combinations of invasive and native plants), pot disturbance (disturbed: substrate in the pot disturbed before target plants inserted; non-disturbed: target plants planted into non-disturbed substrate, resulting in non-disturbed CMNs of M+ pots), and harvest (harvest 1: first harvest; harvest 2: second harvest). Bars represent means accompanied by standard errors (n = 5). Significances of plant species (plant), disturbance, and harvest, and their interaction are indicated (factors with $P \geq 0.05$ are not shown, ***$P < 0.001$, **$0.001 \leq P < 0.01$, *$0.01 \leq P < 0.05$).

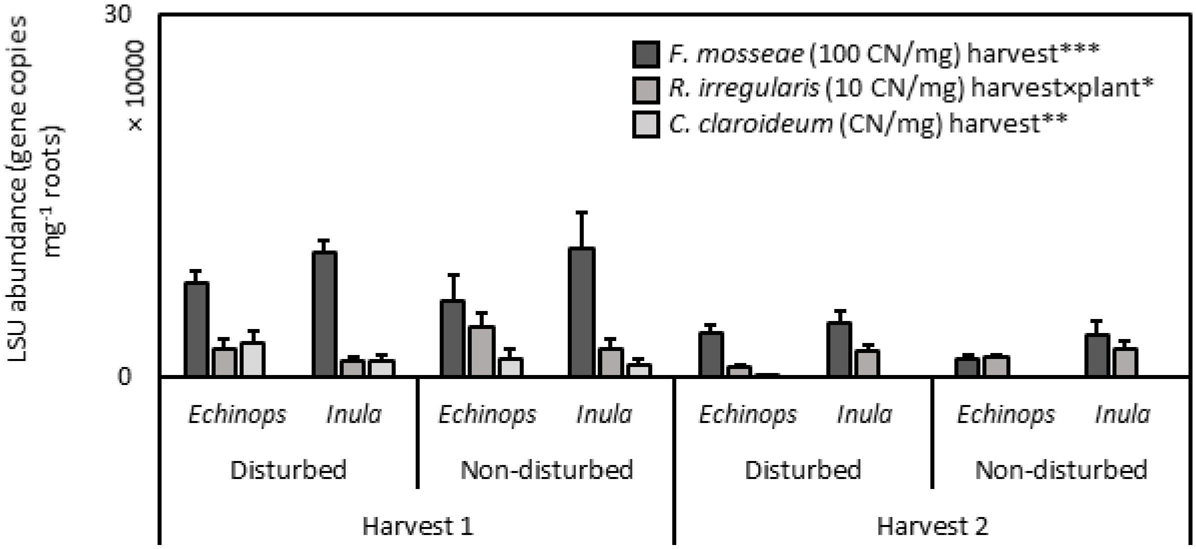

Fig. S2 Shoot dry biomass and shoot P content of the native plant species *Inula conyzae* paired with invasive plant species *Echinops sphaerocephalus* as affected by mycorrhizal inoculation (M+: mycorrhizal inoculum added; M−: nonmycorrhizal control), community assembly i. e., plant combination (invasive-native: invasive and native plant growing in competition; native-native: only native plants of the same species growing together) and initial soil disturbance (disturbed: substrate in the pot disturbed before target plants inserted; non-disturbed: target plants planted into non-disturbed substrate, resulting in non-disturbed CMNs of M+ pots) after the first harvest. Bars represent means accompanied by standard errors (n = 5). Different letters above individual bars within vertical dashed lines indicate significant differences between means at $P < 0.05$.

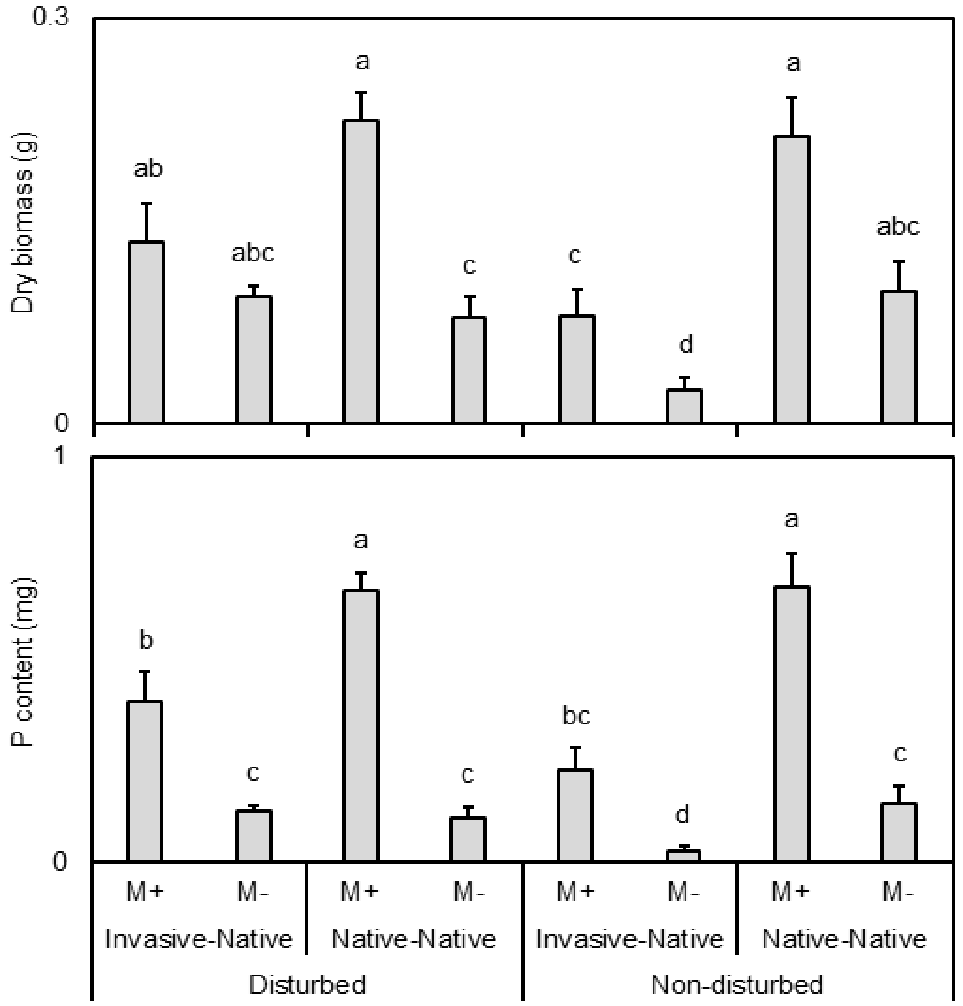

Fig. S3 Shoot dry biomass and shoot P content of the invasive plant species *Echinops sphaerocephalus* when paired with native plant species *Inula conyzae*. Bars represent means accompanied by standard errors (n = 5).

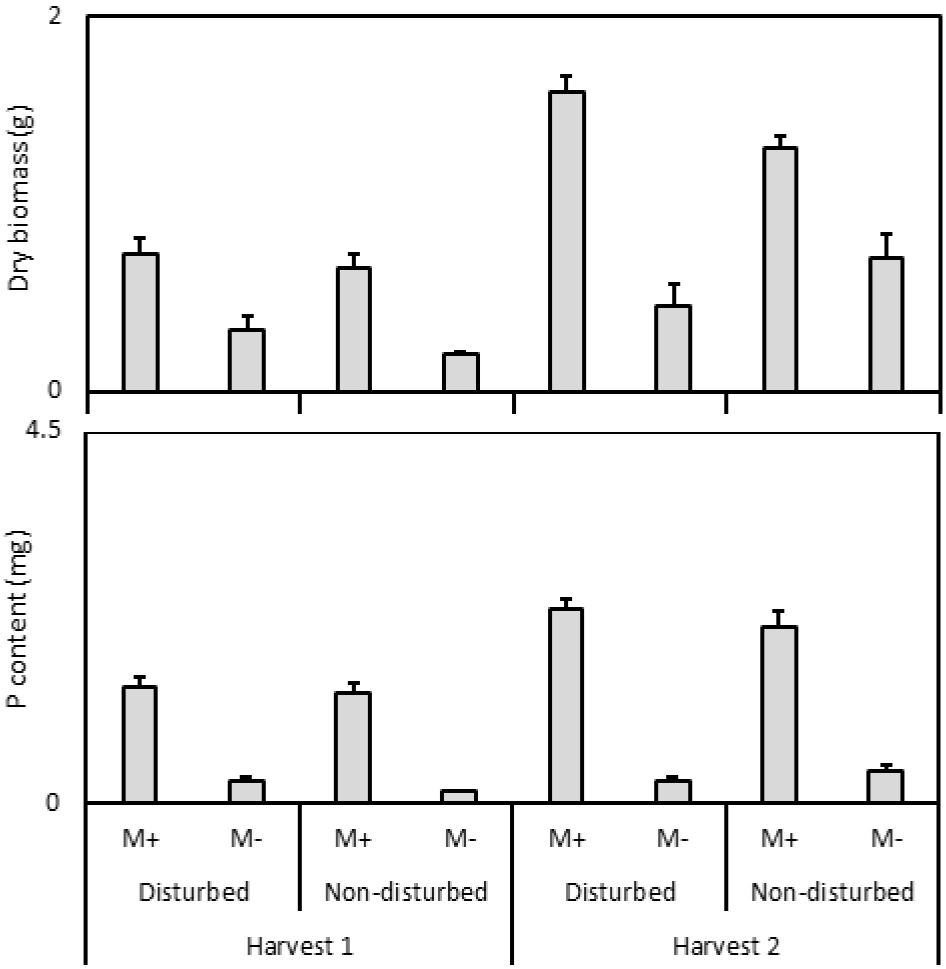

REFERENCES

[1] Thonar C, Erb A, Jansa J (2012) Real-time PCR to quantify composition of arbuscular mycorrhizal fungal communities-marker design, verification, calibration and field validation. Molec Ecol Res 12, 219-232.

Table S6. Raw data table.

Article title: Arbuscular mycorrhizal fungi favor invasive Echinops sphaerocephalus when grown in competition with native Inula conyzae

Authors: Veronika Řezáčová, Milan Řezáč, Hana Gryndlerová, Gail W. T. Wilson and Tereza Michalová

Pot number	Invasive plant species	Harvest	Inoculation	Disturbance	Community assembly	Shoot DW (g) of the left positioned (invasive or native) plant	Shoot DW (g) of the right positioned (always native) plant	Shoot P (mg P g^{-1} DW) of the left positioned (invasive or native) plant	Shoot P (mg P g^{-1} DW) of the right positioned (always native) plant	Shoot DW (g) of the additional plants	LSU abundance of *Claroideoglomus claroideum* (gene copies mg^{-1} roots)	LSU abundance of *Glomus intraradices* (gene copies mg^{-1} roots)	LSU abundance of *Funneliformis mosseae* (gene copies mg^{-1} roots)
1	*Echinops sphaerocephalus*	1	M+	no	invasive-native	0.7346	0.0257	1.918561	2.7844	4.709	0	26 741	41 826
2	*Echinops sphaerocephalus*	1	M+	no	invasive-native	0.8341	0.1002	1.738106	2.638065	3.161	9 338	3 800	30 805
3	*Echinops sphaerocephalus*	1	M+	no	invasive-native	0.7328	0.0446	2.330732	2.824494	3.89	24 591	58 366	134 862
4	*Echinops sphaerocephalus*	1	M+	no	invasive-native	0.4163	0.1453	2.383676	3.062583	3.908	14 131	13 472	215 200
5	*Echinops sphaerocephalus*	1	M+	no	invasive-native	0.5498	0.0861	2.049819	2.628191	4.039	0	8 560	104 952
6	*Echinops sphaerocephalus*	1	M+	no	native-native	0.2172	0.1563	2.973278	3.293265	4.736	11 444	53 494	83 633
7	*Echinops sphaerocephalus*	1	M+	no	native-native	0.3554	0.2943	2.820494	3.551221	2.91	7 264	1 477	131 052
8	*Echinops sphaerocephalus*	1	M+	no	native-native	0.1158	0.1537	3.511047	3.372075	3.823	51 522	89 745	229 374
9	*Echinops sphaerocephalus*	1	M+	no	native-native	0.2215	0.1879	3.09638	3.314526	3.44	55 843	8 915	287 696
10	*Echinops sphaerocephalus*	1	M+	no	native-native	0.2258	0.2001	3.255793	3.064442	3.454	9 711	39 692	176 816

11	*Echinops sphaerocephalus*	1	M+	yes	invasive-native	0.6651	0.1096	1.909701	2.824088	3.19	26 904	10 550	126 933
12	*Echinops sphaerocephalus*	1	M+	yes	invasive-native	0.4954	0.2513	2.164155	2.73668	3.215	0	6 684	69 622
13	*Echinops sphaerocephalus*	1	M+	yes	invasive-native	0.9622	0.1109	1.834843	3.112558	2.893	11 280	15 579	124 571
14	*Echinops sphaerocephalus*	1	M+	yes	invasive-native	0.8804	0.1445	1.868996	3.032404	2.945	22 150	9 346	87 999
15	*Echinops sphaerocephalus*	1	M+	yes	invasive-native	0.6338	0.0563	2.094434	3.431401	3.609	0	21 823	103 875
16	*Echinops sphaerocephalus*	1	M+	yes	native-native	0.1829	0.2678	2.54168	3.064503	3.758	27 755	97 538	179 825
17	*Echinops sphaerocephalus*	1	M+	yes	native-native	0.2471	0.1066	3.177555	3.211667	3.844	0	30 564	105 213
18	*Echinops sphaerocephalus*	1	M+	yes	native-native	0.1372	0.2781	3.295088	3.156728	3.604	73 440	137 650	309 912
19	*Echinops sphaerocephalus*	1	M+	yes	native-native	0.1633	0.24	2.902138	3.339656	3.166	81 388	274 035	154 187
20	*Echinops sphaerocephalus*	1	M+	yes	native-native	0.2465	0.3794	2.931507	2.582032	3.07	20 732	115 493	148 569
21	*Echinops sphaerocephalus*	1	M-	no	invasive-native	0.2566	0.0639	0.793496	1.263107	3.837			
22	*Echinops sphaerocephalus*	1	M-	no	invasive-native	0.1703	0.0104	0.585235	0.478142	4.261			
23	*Echinops sphaerocephalus*	1	M-	no	invasive-native	0.1694	0.0111	0.800062	0.720315	4.79			
24	*Echinops sphaerocephalus*	1	M-	no	invasive-native	0.1967	0.0238	0.589478	1.181435	4.224			
25	*Echinops sphaerocephalus*	1	M-	no	invasive-native	0.1886	0.0142	0.664782	0.798792	3.946			
26	*Echinops sphaerocephalus*	1	M-	no	native-native	0.065	0.2933	1.655701	1.65027	3.005			
27	*Echinops sphaerocephalus*	1	M-	no	native-native	0.1694	0.0732	1.798666	1.586529	3.481			
28	*Echinops sphaerocephalus*	1	M-	no	native-native	0.0414	0.1343	1.429218	0.936988	3.372			
29	*Echinops sphaerocephalus*	1	M-	no	native-native	0.0247	0.0905	1.206286	1.600764	3.721			
30	*Echinops sphaerocephalus*	1	M-	no	native-native	0.0482	0.0397	1.145996	1.093882	3.448			
31	*Echinops sphaerocephalus*	1	M-	yes	invasive-native	0.132	0.0666	0.73911	1.50582	3.611			
32	*Echinops sphaerocephalus*	1	M-	yes	invasive-native	0.3984	0.1061	0.76266	1.378286	3.224			
33	*Echinops sphaerocephalus*	1	M-	yes	invasive-native	0.3468	0.1227	0.744422	1.474127	3.258			
34	*Echinops sphaerocephalus*	1	M-	yes	invasive-native	0.5636	0.0916	0.818635	1.107234	3.604			
35	*Echinops sphaerocephalus*	1	M-	yes	invasive-native	0.1994	0.0825	0.861635	1.283103	2.766			
36	*Echinops sphaerocephalus*	1	M-	yes	native-native	0.0472	0.0924	1.036002	1.323853	3.504			
37	*Echinops sphaerocephalus*	1	M-	yes	native-native	0.0777	0.1637	1.609906	1.18554	2.88			
38	*Echinops sphaerocephalus*	1	M-	yes	native-native	0.1816	0.0316	1.81165	1.161182	3.08			
39	*Echinops sphaerocephalus*	1	M-	yes	native-native	0.045	0.1065	1.189114	1.467235	3.607			
40	*Echinops sphaerocephalus*	1	M-	yes	native-native	0.0208	0.0233	0.670852	0.66488	4.144			
121	*Echinops sphaerocephalus*	2	M+	no	invasive-native	1.1341	0.1659	1.667863	2.816574	5.137	0	10 019	13 546
122	*Echinops sphaerocephalus*	2	M+	no	invasive-native	1.4172	0.242	1.978187	2.586165	3.858	0	48 150	35 484
123	*Echinops sphaerocephalus*	2	M+	no	invasive-native	1.223	0.2223	1.45502	2.847394	4.269	0	8 979	20 830
124	*Echinops sphaerocephalus*	2	M+	no	invasive-native	1.4791	0.2895	1.673207	2.430097	3.76	0	20 874	17 169
125	*Echinops sphaerocephalus*	2	M+	no	invasive-native	1.2235	0.3071	1.465367	2.836192	3.983	0	25 241	82 693
126	*Echinops sphaerocephalus*	2	M+	no	native-native	1.0039	0.453	2.7493	2.722238	3.608	0	47 637	13 921

127	*Echinops sphaerocephalus*	2	M+	no	native-native	0.3435	1.1869	2.606807	2.906865	4.137	0	22 120	35 367
128	*Echinops sphaerocephalus*	2	M+	no	native-native	0.5654	1.1289	3.141934	3.46427	4.332	0	39 967	18 478
129	*Echinops sphaerocephalus*	2	M+	no	native-native	0.6387	0.7996	2.724625	3.199318	4.334	10 134	98 618	35 673
130	*Echinops sphaerocephalus*	2	M+	no	native-native	0.6589	0.9028	3.256827	3.295505	3.877	0	93 368	77 604
131	*Echinops sphaerocephalus*	2	M+	yes	invasive-native	1.6157	0.0711	1.563122	2.194356	4.262	0	784	14 438
132	*Echinops sphaerocephalus*	2	M+	yes	invasive-native	1.3419	0.2692	1.432372	2.577573	3.71	0	23 036	28 410
133	*Echinops sphaerocephalus*	2	M+	yes	invasive-native	1.4628	0.3926	1.464858	2.927699	3.928	0	36 266	74 904
134	*Echinops sphaerocephalus*	2	M+	yes	invasive-native	1.7965	0.0825	1.313181	2.338566	3.817	0	25 334	43 672
135	*Echinops sphaerocephalus*	2	M+	yes	invasive-native	1.7417	0.1768	1.613263	2.436501	4.305	0	20 373	61 088
136	*Echinops sphaerocephalus*	2	M+	yes	native-native	0.7704	0.7997	3.001866	2.7538	3.132	0	151 422	32 946
137	*Echinops sphaerocephalus*	2	M+	yes	native-native	0.5307	0.7046	3.039482	3.341626	3.638	0	124 562	51 721
138	*Echinops sphaerocephalus*	2	M+	yes	native-native	0.7588	0.8353	2.852078	2.625359	4.574	0	212 235	53 903
139	*Echinops sphaerocephalus*	2	M+	yes	native-native	1.0619	0.9106	2.639744	2.986276	3.799	42 175	169 064	160 849
140	*Echinops sphaerocephalus*	2	M+	yes	native-native	0.8775	0.9262	2.631825	3.225611	4.426	88 566	477 622	175 851
141	*Echinops sphaerocephalus*	2	M-	no	invasive-native	0.508	0.16	0.667406	1.661568	3.974			
142	*Echinops sphaerocephalus*	2	M-	no	invasive-native	0.8418	0.2909	0.594767	1.539581	2.675			
143	*Echinops sphaerocephalus*	2	M-	no	invasive-native	0.5383	0.4166	0.462829	1.46218	3.587			
144	*Echinops sphaerocephalus*	2	M-	no	invasive-native	0.4951	0.2199	0.533837	1.648889	4.155			
145	*Echinops sphaerocephalus*	2	M-	no	invasive-native	1.17	0.421	0.517536	1.430343	3.139			
146	*Echinops sphaerocephalus*	2	M-	no	native-native	0.1409	0.2147	1.720133	1.776236	3.908			
147	*Echinops sphaerocephalus*	2	M-	no	native-native	0.2375	0.1579	1.48226	1.680201	3.676			
148	*Echinops sphaerocephalus*	2	M-	no	native-native	0.2021	1.1579	1.630908	1.633927	1.547			
149	*Echinops sphaerocephalus*	2	M-	no	native-native	0.1158	0.2065	1.699029	1.532985	3.894			
150	*Echinops sphaerocephalus*	2	M-	no	native-native	0.4214	0.4459	1.528855	1.513823	3.485			
151	*Echinops sphaerocephalus*	2	M-	yes	invasive-native	0.1365	0.2977	0.62935	1.758487	3.631			
152	*Echinops sphaerocephalus*	2	M-	yes	invasive-native	0.2964	0.2301	0.484923	1.507796	3.741			
153	*Echinops sphaerocephalus*	2	M-	yes	invasive-native	0.7851	0.2314	0.636276	1.528672	2.15			
154	*Echinops sphaerocephalus*	2	M-	yes	invasive-native	0.6345	0.2838	0.486679	1.641451	3.967			
155	*Echinops sphaerocephalus*	2	M-	yes	invasive-native	0.4306	0.3082	0.632766	1.543955	3.735			
156	*Echinops sphaerocephalus*	2	M-	yes	native-native	0.1687	0.2692	1.661469	1.643517	4.671			
157	*Echinops sphaerocephalus*	2	M-	yes	native-native	0.84	0.3521	1.355623	1.632749	3.64			
158	*Echinops sphaerocephalus*	2	M-	yes	native-native	0.505	0.4409	1.629025	1.61933	3.3			
159	*Echinops sphaerocephalus*	2	M-	yes	native-native	0.1538	0.8111	1.601985	1.534301	3.155			
160	*Echinops sphaerocephalus*	2	M-	yes	native-native	0.5355	0.4885	1.535889	1.584315	2.734			
